# Good practices in collecting umbilical cord and placental blood**^1^**

**DOI:** 10.1590/1518-8345.0781.2770

**Published:** 2016-08-18

**Authors:** Lauren Auer Lopes, Elizabeth Bernardino, Karla Crozeta, Paulo Ricardo Bittencourt Guimarães

**Affiliations:** 2 MSc, RN, Complexo do Hospital de Clínicas, Universidade Federal do Paraná, Curitiba, PR, Brazil.; 3 PhD, Adjunct Professor, Departamento de Enfermagem, Universidade Federal do Paraná, Curitiba, PR, Brazil.; 4 PhD, Adjunct Professor, Departamento de Estatística, Universidade Federal do Paraná, Curitiba, PR, Brazil.

**Keywords:** Nursing, Practice Management, Blood Banks, Stem Cells, Fetal Blood

## Abstract

**Objective:**

to identify the factors related to the quality of umbilical cord and placental blood specimens, and define best practices for their collection in a government bank of umbilical cord and placental blood.

**Method:**

this was a descriptive study, quantitative approach, performed at a government umbilical cord and placental blood bank, in two steps: 1) verification of the obstetric, neonatal and operational factors, using a specific tool for gathering data as non-participant observers; 2) definition of best practices by grouping non-conformities observed before, during and after blood collection. The data was analyzed using descriptive statistics and the following statistical software: Statistica^(r)^ and R^(r)^.

**Results:**

while there was a correlation with obstetrical and neonatal factors, there was a larger correlation with operational factors, resulting in the need to adjust the professional practices of the nursing staff and obstetrical team involved in collecting this type of blood. Based on these non-conformities we defined best practices for nurses before, during and after blood collection.

**Conclusion:**

the best practices defined in this study are an important management tool for the work of nurses in obtaining blood specimens of high cell quality.

## Introduction

According to ANVISA (Brazil's National Health Surveillance Agency), umbilical cord and placental blood (UCPB) may be collected by a nurse or other duly qualified university-level technician during the third stage of a normal or Cesarean delivery^1^. The goal of the procedure is to collect hepatopoietic stem cells (HSCs) for government umbilical cord and placental blood banks (UCPBBs), for subsequent used for transplantation and for the treatment of a number of malignant and non-malignant diseases^2^.

Quality assurance of the HSCs collected depends directly on the performance of the obstetrical team professionals. For this reason, cellularity has been the focus of attention of the nursing staff, because of the need to collect a suitable volume of HSCs to supply the UCPBBs.

Thus, awareness of the obstetric, neonatal and operational factors that can influence the volume and cellularity of the UCPB specimens must be included among the numerous competences of nurses working in the UCPBBs. Knowing what these factors are, enables defining best practices for collecting a suitable volume of UCPB specimens with adequate cellularity, contributing to satisfactory outcomes in a number of different situations and contexts^5^. The term "best practices" has been widely used in healthcare since 1990, starting with a discussion on tools to manage quality in healthcare institutions, sponsored by the Pan-American Health Organization^6^.

This study attempts to identify factors related to the quality of UCPB specimens, and to define best practices for their collection within a UCPBB.

## Method

A descriptive survey using a quantitative approach, conducted at an off-site HSC collection center of the UCPBB of a university hospital located in the south of the country. A nurse is responsible for managing the service at the study location, for selecting and attracting women to donate UCPB specimens, for harvesting the stem cells, entering the data into the national umbilical cord stem-cell registry (Renacord), shipping the specimens, for populating production and quality indicators, and for follow-up visits. We reiterate that in this service all UCPB specimens are collected by nurses.

This study was conducted in two steps: 1a) Verification of the obstetric, neonatal and operational factors, using a specific data collection tool and observing as a non-participants; 2a) definition of best practices by grouping non-conformities observed before, during and after UCPB collection.

This study complies with the ethical standards of National Board of Health (CNS) Resolution 466/2012, and was approved by the institution's Research Ethic Committee, as per the Opinion n. 327,621 in *Plataforma Brasil*, and by the Brazilian National Research Ethics Committee (CONEP) on August 7 2013, under number 16117713.1.0000.0102. Participants were informed of the study procedure and signed a Free and informed Consent Form (FICF).

Study participants were split into two groups:

-50 voluntary UCPB donors, recruited between August 2013 and April 2014;
-14 professionals from the obstetrical team, who validated best practices in UCPB collection between September and October 2014.

The first step in the study consisted of checking obstetrical, neonatal and operational factors. Data was obtained by analyzing the files of the 50 volunteer mothers who donated HSCs, and of their neonates, the UCPBB entries for material and neonate factors, and the records of a field diary originating from non-participant observations (a total of 12 hours and 50 minutes) of placental processing for operating factors.

We used a semi-structured data collection tool designed based on the historical data on the output of the study location, identifying the causes and interferences in the disposal of UCPB specimens at that service, as well as a review of the current literature on the factors that influence volume and cellularity. Content validation was obtained from nurses and experts working in other UCPBBs of the Brasil Cord Network, using the Delphi Technique. The criteria for selecting these experts included nurse qualification and experience^7^, and the history of UCPBB production. We selected four nurses, but only three fulfilled the inclusion criteria. We conducted three rounds, with "agree" and "disagree" options for each item of the questionnaire, and the option "considerations" for other contributions provided by the nurses. Following the third round and after reaching a consensus, we decided we had the final data collection questionnaire for the first step of the study.

For quality of specimens we defined a minimum cellularity of 7x10^8^ of total nucleated cells for UCPB collection, considering the value used by the UCPBB in question.

The data collected during this step was tabulated and systematized using an Excel spreadsheet, and submitted to descriptive quantitative statistical analyses, and to content analyses by approximation before being processed using *^_R(r)_^* and*^_Statistica(r)_^* software*.*

Initially, the data was summarized by calculating descriptive measures, means, minimums, maximums and standard deviations for continuous variables, and frequency tables for the categorical variables. To identify the reasons that UCPB specimens had been discarded, we applied non-parametric association statistical techniques, based on the assumption that data normality had not been achieved, using Lilliefors's adherence test. To check the association between continuous variables we used Spearman's coefficient, with 5% as the significance level (p<0.05). For the categorical variables (comparison of two independent samples, we used Mann-Whitney or Kruskal-Wallis (comparison between more than two independent samples). We adjusted the multiple regression models for the variables "cellularity" and "volume" using a Stepwise procedure.

Based on the data collected in the first step, we prepared a list of best practices for this UCPBB, considering the results of the obstetrical, neonatal and operational factors we analyzed. This list was grouped by the researchers into three categories: before, during and after UPCB collection, and then submitted for analysis and consensus at expert meetings. Fourteen obstetrics professionals participated in these meetings (obstetricians, residents in obstetrics, professors of obstetrics and nurses). This group met for a total of 19 hours and 20 minutes of one-on-one discussions. Content validation by the experts included the assessment of the overall list of good practices and individual actions (clarity and relevance)^7^. There was no disagreement with any of the items submitted, thus the list of good practices for UCPB collection at this UCPBB was validated.

## Results

### Factors influencing volume and cellularity of umbilical cord and placental blood specimens

The following obstetrical factors influence volume and cellularity: placental weight: volume (*rs*=0.382923, *p*=0.006057) and cellularity (*rs=*0.339155, *p*=0.015978); characteristics (diameter) of the umbilical cord: volume (SD± 29.56, *p*=0.0051) and cellularity (SD± 4.86, *p*=0.0095); blood flow: volume (SD± 29.56, *p*=0.00001) and cellularity (SD± 3.64, *p*=0.00001), showing that the heavier the placenta and the wider and fuller the umbilical cord, the larger the amount of UCPB collected and the cellularity of the specimens.

The only neonatal factor identified was weight of the newborn, which was found to be directly associated with volume (*rs*=0.362558, *p*=0.009667), but had no influence on cellularity, showing that larger birth-weight is associated with a greater volume of HSC collected.

*Operational factors* were found to have the greatest influence on HSC cellularity, with a direct relationship between volume collected and cellularity *(rs*=0.873151, *p*=0.000000). The more blood collected, the greater the cellularity.

Collecting a suitable volume of UCPB is related to incidents during placental handling by the obstetrics professional, such as inadequate clamping and cord sectioning (leaving the segment short - less than 30 cm), which was found in 29.37% of the collections (SD ± 19.96, *p*=0.00054), excess manual pressure during placental elimination with controlled traction (23.43% of the collections) (SD± 10.87, *p*=0.0004); rupture or laceration of the cord segment due to excessive manual pressure (21.60% of the collections) (SD± 10.80, *p*= 0.021); and the obstetrical team forgetting to clamp the cord segment (14.80% of the collections) (SD± 5.81, *p*= 0.0007). The relationship with operational factors indicates that inadequate handling of the placenta results in collecting less voluminous UCPB specimens.

These and other factors showed a significant association with the cellularity of the UCPB specimens: inadequate cord section (leaving the segment too short - under 30 cm) (5.26% of the collections) (SD± 19,96, *p*=0,00054), excess manual pressure during placental elimination with controlled traction (23.43% of the collections) (SD± 10.87, *p*=0.0004); rupture or laceration of the cord segment due to excessive manual pressure (21.60% of the collections) (SD± 10.80, *p*= 0.021); and the obstetrical team forgetting to clamp the cord segment (14.80% of the collections) (SD± 5.81, *p*= 0.0007). These factors contribute to low volume of collection and thus low cellularity of the UCPB specimen.

We also found a correlation between improper placental handling and the volume obtained and the appearance of the cord segment, as 45.52% of the cords were macerated (SD± 29.56, *p*=0.0003), which influenced the cellularity of 8.13% of the units (SD± 4.86, *p*=0.0001). It is supposed that maceration of the collected cords was due to excessive manual pressure during placental expulsion by controlled traction, and the sequence of clamping of the umbilical cord segments.

Another operational factor we found to influence quality was the time taken between collecting the UCPB and shipping and processing the collected specimen. This was related to volume (SD± 29.56, *p*=0.0355) and final cellularity (SD± 4.86, *p*=0.0233). Of the 50 specimens collected, 44 were processed within a total of 12 hours or less, and only 6 in 24 hours or less.

We found better final laboratory volume (Mean=42.39545, SD± 28.42352) and cellularity (Mean=7.58409, SD± 4.794907) yields for specimens processed within 12 hours, compared to those processed within 24 hours (mean volume=68.45, SD± 30.02957, mean cellularity =12.13333, SD± 3.488075). Thus the longer the time between UCPB collection, shipment and processing, the lower the final blood volume and cellularity.

This study demonstrated the obstetric, neonatal and operational factors that influenced the volume and cellularity of the 50 blood specimens collected. Only 18 had sufficient volume and cellularity to be cryopreserved. The remaining 32 were disqualified due to low volume (SD± 29.56, *p*=0.00001) or cellularity (SD± 4.86, *p*=0.00001), resulting in 64% disqualification.

### Non-conformities in collecting umbilical cord and placental blood

Based on our analysis of the results of the first step of the study, we found the obstetrical team often mishandles the placenta, making it unsuitable for collecting UCPB by the UCPBB nurses, in particular the following non-conformities:

- Inadequate clamping and sectioning of the umbilical cord segment;
- Excess manual pressure during placental expulsion using controlled traction;
- Rupture or laceration of the umbilical cord segment;
- Unclamping of the umbilical cord segment (because it was forgotten, to collect specimens or empty the placenta);
- Cord segment clamping sequence during controlled traction to eliminate the placenta.

In addition to the non-conformities listed, unsatisfactory performance by UCPBB nurses in managing the time taken to collect, ship and process UCPB specimens.

Thus, based on the non-conformities and factors of influence identified, and the responsibilities of UCPBB nurses, we defined nursing best practices before, during and after collection. These were validated by the current UCPBB obstetrical team.

### Good practices in collecting umbilical cord and placental blood:

a) Before collecting umbilical cord and placental blood:

- Gather data on potential UCPB donors in the Obstetrical Surgical Center. The obstetrical team should report incidents that would disqualify the UCPB donation.
- Assess the physiological (pain threshold) and emotional status of candidates for donation before they are approached by the nurse for an interview and signature of the FICF.
- Signal a potential donor on her file and inform the obstetrical team of the possibility that extra-uterine UCPB will be collected after placental expulsion;
- Keep all of the collection material ready and easy to reach before the placenta is placed in the hands of the obstetric professional;
- Follow the labor of each potential UCPB donor;
- Remove candidates from the donation list if there are simultaneous deliveries, preferring to collect material from candidates where ultrasound indicates higher birth-weight, from larger and heavier placentas (where data is available), and from the wider umbilical cords with greater apparent blood flow;
- Monitor and instruct the obstetrical team during clamping, sectioning the umbilical cord and placenta, and placental expulsion.

b) When collecting umbilical cord and placental blood:

- Place the placenta in the placental support, being careful not to pinch the vessels in the umbilical cord segment in the window;
- Use the two needle extremities of the closed collection system, replacing them in the event of clots;
- Milk the placenta manually when the last vessel in the cord segment is punctured, making sure to use the proper technique so as not to contaminate the closed collection system.

c) After collecting umbilical cord and placental blood:

- Forward the UCPB units collected to the processing lab, preferably on the same day and always within less than 24 hours;
- Maintain a continuous education program to train the obstetrical team and new nurses.

## Discussion

*Obstetric and neonatal factors, "*placental weight" and "newborn weight" were also found in other studies to influence SCUP volume and cellularity^5^ ^,^ ^8^ ^-^ ^9^. However, there is no indication of minimum or maximum placental weight for harvesting UCPB specimens. No studies were found on the obstetrical factor "characteristics of the umbilical cord segment" (diameter and blood flow).

On the influence of *operational factors* related to handling the placenta on UCPB volume and cellularity, there is evidence that incorrect sectioning of the umbilical cord (less than five centimeters from the abdomen)^2^ results in smaller volumes and cellularity^8^ ^-^ ^10^.

Knowledge of other operating factors related to inadequate handling of the placenta are innovative to the practice of nursing at UCPBB, given that there are no records of any literature on this theme. Although not all of the factors described are actionable by the nurse, it is worth reiterating that they have a direct influence on promoting and performing UCPB collections, given that inadequate placental handling results in low collection volume, and thus low cellularity of the UCPB specimen. For this reason, they were considered when defining best practices for UCPB collection.

The time between collecting the SCUP, transporting and processing the unit was considered here to be an operational factor, and depends largely on the professional performance of the nurse. The nurse is responsible for the logistics of adequately transporting units for early processing. It is known that delaying the processing of blood specimens results in a gradual decrease in the number of nucleated, viable and CD34+ cells, thus time between collection and processing should be minimized^11^.

Given the results presented herein, we defined a list of best practices to collect SCUP, given that there is not much scientific evidence for collecting SCUP. 

An evidence-based practice could be a great ally to the UCPBB nurse, helping promote service excellence. Regarding the object of this study, we found that the nurse is responsible for selecting and attracting UCPB donors, for collecting specimens, donor serology, nursing management and donor follow-up. Therefore, the nurse must be vigilant regarding the different factors that can interfere in the operation of UCPB harvesting and storage in order to comply with all recommendations and apply the best practices available to get suitable SCUP cellularity and volume.

In addition to being aware of the factors that influence SCUP volume and cellularity, the technical competence of the nurse, his or her interaction with the obstetrical team and the collaboration between them are essential for a successful harvest. It is considered good practice for the nurse to keep a training program for the obstetrical team to optimize donor selection^12^ and ensure proper placental handling.

Among the best practices tools available to the nurse, we highlight communication and planning. Communication, as an articulator, is essential for nursing management and to enable the work^13^ of all of the professionals directly or indirectly involved in UCPB specimen collection. Planning activities is essential for the proper harvesting of UCPB ^(^ ^14^, ensuring the process is efficient, practical and safe.

Thus, adopting good practices in UCPB assumes a change in attitude and actions on the individual, collective and organizational level, implying in potential benefits for the care provided^6^.

## Conclusion

This study identified factors related to the quality of UCPB specimens, and defined best practices for their collection within an UCPBB.

Considering that at the study location specimen collection is done exclusively by nurses, finding factors that have a positive influence on volume and cellularity is essential for defining professional practices for obtaining high quality blood specimens.

This study shows the need to develop and improve the technical skills of the obstetrical team when handling the placenta in the third stage of delivery to harvest SCUP. In this process, the role of the nurse in operations and service management is strategic for the UCPBB healthcare team.

The study conducted at a single UCPBB, and the possibility of validating good practices by the service team could be considered limitations for this study. However, given the shortage of studies in this area, and the wealth of information, the data obtained can foster new studies or contribute to defining good practices to be adopted by other UCPBB. In the same way, the good practices listed could serve as a guide to be used by obstetrical professionals, especially those who are starting as resident physicians or nurses, as the results of this study were obtained at a teaching hospital.
